# Diagnosis of pulmonary *Mycobacterium heckeshornense* infection using matrix-assisted laser desorption ionization time-of-flight mass spectroscopy

**DOI:** 10.1016/j.idcr.2021.e01296

**Published:** 2021-09-23

**Authors:** Hayato Kinoshita, Mariko Ono, Yoko Nagatomo, Yukihisa Takeda, Hiroyuki Nakamura, Kazutetsu Aoshiba

**Affiliations:** a Department of Respiratory Medicine, Tokyo Medical University Ibaraki Medical Center, Ibaraki 300-0395, Japan; b Department of Respiratory Medicine, Tokyo Medical University, 6-7-1 Nishishinjuku, Shinjuku-ku, Tokyo 160-0023, Japan

A 64-year old man presented to our hospital with a 1-year history of productive cough. He had a smoking history of 60 packs/year. Chest X-ray and computed tomography (CT) scan revealed fibrocavitary lesions surrounded by emphysematous changes in the bilateral upper lobes (Fig. 1). The direct acid-fast bacilli (AFB) stain test of the sputum was positive, although polymerase chain reaction tests for *Mycobacterium tuberculosis* and Mycobacterium avium complex were negative. AFB were cultured in the mycobacterium growth indicator tube (MGIT) systems and *Mycobacterium heckeshornense* (*M. heckeshornense*) was identified using matrix-assisted laser desorption ionization time-of-flight mass spectroscopy (MALDI-TOF MS). Treatment with rifampicin and ethambutol was initiated, but it was discontinued 17 days later because of the development of cutaneous drug eruptions. Lately obtained results of indirect drug susceptibility test showed that the isolate was susceptible to many antimycobacterial drugs (Table 1). Although the AFB stain test and mycobacterium cultures of sputum samples remained positive, the patient’s clinical conditions and chest CT findings (Fig. 1) were stable without further treatment with antibiotics treatment during the 2-year follow-up period.

**Fig. 1:**
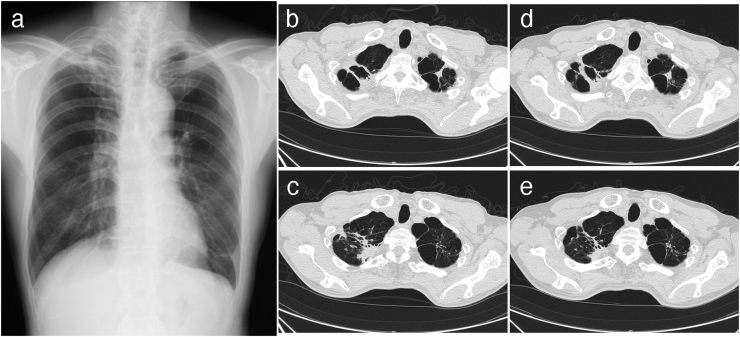
Images of chest X-ray and computed tomography (CT) scan. (a) Chest X-ray at the initial visit shows patchy opacifications with fibrotic shadows in the upper right lung. (b and c) Chest CT scan taken at the initial visit shows fibrocavitary lesions surrounded by emphysematous changes on the right side predominantly in the bilateral upper lobes. These lesions remained stable after 2 years of follow-up (d and e).

**Table 1:** Results of the drug susceptibility test.

Drug	Minimal inhibitory concentration (mg/L)	Interpretation
Amikacin	≤0.5	Susceptible
Clarithromycin	≤0.03	Susceptible
Ethambutol	≤0.06	Susceptible
Kanamycin	≤0.06	Susceptible
Levofloxacin	≤0.03	Susceptible
Rifabutin	0.015	Susceptible
Rifampin	≤0.03	Susceptible
Streptomycin	≤0.06	Susceptible
Ethionamide	2	Intermediate

*M. heckeshornense* is a rare, slow growing, scotochromogenic organism that was firstly isolated from patients with pulmonary infection [1]. Previous cases of *M. heckeshornense* infection have reported the involvement of the lungs, bones, muscles, and lymph nodes [1], [2], [3], [4], [5]. The diagnosis of *M. heckeshornense* infection is somewhat problematic. First, *M. heckeshornense* has a very slow growth rate [1] and often grows only in MGIT, not on solid medium [4]. Second, *M. heckeshornense* can be misidentified as *Mycobacterium. xenopi* in a commercially available DNA–DNA hybridization kit [3], [5]; therefore, DNA sequence analysis of 16S rRNA, *rpo*B, and *hsp*65 is required for identification. However, MALDI-TOF MS systems have started being used commercially and are reliable in identifying *M. heckeshornense* species [3], [4]. Some previous studies have reported that *M. heckeshornense* is resistant to isoniazid but susceptible to rifampicin, ethambutol, clarithromycin, streptomycin, and levofloxacin [4], [5].

Despite persistent sputum isolation of the species and discontinuation of the short-course antibiotic treatment due to cutaneous drug eruptions, the patient reported in the present study remained stable. In parallel with our case report, previously reported radiographic findings of pulmonary *M. heckeshornense* infection include upper lobe-dominant locations of cavitary, nodular, and/or infiltrative lesions [4], which make it difficult to distinguish from infection caused by other *Mycobacterium* species. However, MALDI-TOF MS is useful for the diagnosis of *M. heckeshornense* infection.

## Ethical approval

This case report meets the standards of the Tokyo Medical University Ethics Committee.

## Consent

Written informed consent was obtained from the patient for publication of this case report and accompanying images. A copy of the written consent is available for review by the Editor-in-Chief of this journal on request.

## Funding

This research did not receive any specific grant from funding agencies in the public, commercial, or not-for-profit sectors.

## Authors' contributions

All authors have made significant contributions to the planning, conduct, and reporting of the work described in this article. All authors have read and approved the submission of this final manuscript.

## CRediT authorship contribution statement

**Hayato Kinoshita:** Writing – original draft. **Mariko Ono:** Writing – review & editing. **Yoko Nagatomo:** Writing – review & editing. **Yukihisa Takeda:** Writing – review & editing. **Hiroyuki Nakamura:** Writing – review & editing. **Kazutetsu Aoshiba:** Writing – original draft, Writing – review & editing, Supervision.
